# Anti-Biofilm Activity of Laurel Essential Oil against *Vibrio parahaemolyticus*

**DOI:** 10.3390/foods12193658

**Published:** 2023-10-03

**Authors:** Wenxiu Zhu, Jiaxiu Liu, Yue Zou, Shugang Li, Dongyun Zhao, Haisong Wang, Xiaodong Xia

**Affiliations:** 1 State Key Laboratory of Marine Food Processing and Safety Control, National Engineering Research Center of Seafood, School of Food Science and Technology, Dalian Polytechnic University, Dalian 116034, China; 18742058310@163.com (W.Z.); liujx94@163.com (J.L.); 1717010103@xy.dlpu.edu.cn (Y.Z.); lishugang688@163.com (S.L.); 211710832000922@xy.dlpu.edu.cn (D.Z.); 2 Liaoning Key Lab of Lignocellulose Chemistry and BioMaterials, Liaoning Collaborative Innovation Center for Lignocellulosic Biorefinery, College of Light Industry and Chemical Engineering, Dalian Polytechnic University, Dalian 116034, China; wanghs@dlpu.edu.cn

**Keywords:** *V. parahaemolyticus*, laurel essential oil, biofilm formation, gene expression, motility

## Abstract

*Vibrio parahaemolyticus* is a primary seafood-associated pathogen that could cause gastroenteritis. It can attach to various surfaces and form a biofilm, which poses serious threats to food safety. Hence, an effective strategy is urgently needed to control the biofilm formation of *V. parahaemolyticus*. Laurel essential oil (LEO) is used in food, pharmaceutical and other industries, and is commonly used as a flavoring agent and valuable spice in food industries. The potential antibiofilm effects of LEO against *V. parahaemolyticus* were examined in this study. LEO obviously reduced biofilm biomass at subinhibitory concentrations (SICs). It decreased the metabolic activity and viability of biofilm cells. Microscopic images and Raman spectrum indicted that LEO interfered with the structure and biochemical compositions of biofilms. Moreover, it also impaired swimming motility, decreased hydrophobicity, inhibited auto-aggregation and reduced attachment to different food-contact surfaces. RT-qPCR revealed that LEO significantly downregulated transcription levels of biofilm-associated genes of *V. parahaemolyticus*. These findings demonstrate that LEO could be potentially developed as an antibiofilm strategy to control *V. parahaemolyticus* biofilms in food industries.

## 1. Introduction

*Vibrio parahaemolyticus* (*V. parahaemolyticus*) is a rod-shaped, Gram-negative halophilic bacterium, which is highly abundant in estuarine and coastal waters. *V. parahaemolyticus* is recognized as an important foodborne pathogen transmitted mainly through contaminated seafood throughout the world [1]. The major clinical symptoms of *V. parahaemolyticus* infection include diarrhea, vomiting, chills, fever, nausea and abdominal cramps [2]. Concerns have arisen about a possible increase in the incidence of *V. parahaemolyticus* infection because of increased international trade in seafood, consumption of raw or uncooked seafood, and the number of susceptible populations [3]. Since 1996, outbreaks of infections caused by *V. parahaemolyticus* have occurred worldwide [4]. A large-scale foodborne *V. parahaemolyticus* outbreak associated with raw oyster consumption has occurred in the United States [5]. In Korea, more than 30% of gastroenteritis cases were due to *V. parahaemolyticus* infections during the summer [6]. In the United States, *V. parahaemolyticus* has caused 45,000 cases each year [7]. From 2010 to 2014, 71 *V. parahaemolyticus*-associated outbreaks were recorded in Zhejiang Province, one of the coastal provinces in China, leading to 933 illnesses and 117 hospitalizations [8].

Biofilms are sedentary communities and have a predominantly microbial lifestyle in nature. Biofilms increase bacterial resistance to adverse environments better than cells in planktonic states, which make them difficult to be eradicated. Furthermore, the inherent resistance of biofilms to host immune attacks and conventional antimicrobial agents is an important cause of chronic bacterial infections [9]. *V. parahaemolyticus* can adhere to food-contact surfaces made of various materials (polyurethane, glass, stainless steel, nitrile butyl rubber, etc.), aquatic products (crab, fish, shellfish, shrimp, etc.) and aquaculture equipment to form biofilms [10,11]. Bacterial biofilms have become a serious issue in food industries because biofilms on food-contact surfaces or raw materials may be sources of product spoilage or contamination with pathogenic microorganisms, which can lead to cross contamination and foodborne disease [12]. Hence, developing novel strategies and interventions is imperative to control *V. parahaemolyticus* biofilms in the seafood industry [13].

Essential oils (EOs) are various volatile compounds that are collected from plants. They have highly aromatic properties and possess antibacterial and antioxidant properties [14]. Therefore, EOs have been regarded as food bio-preservatives and widely applied in food industries [15]. The laurel, an evergreen shrub or tree, is indigenous to southern Europe and the Mediterranean region and is extensively cultivated in numerous nations. According to Merghni et al., the primary bioactive components found in laurel consist of 1.8-cineole, α-terpinyl acetate and methyl eugenol. Moreover, 1.8-cineole is frequently utilized as a food flavoring agent in the food industry [16]. Besides, laurel essential oil (LEO) is derived from its leaves and is usually utilized in the food and culinary industries as a flavoring agent and valuable spice [17]. LEO has been proved to have antifungal activities and antibacterial activities [18,19]. However, the anti-biofilm efficacy of LEO against *V. parahaemolyticus* remains unexplored.

The aim of this work is to explore the anti-biofilm effect of LEO against *V. parahaemolyticus*. Meanwhile, the effect of LEO on the metabolic activity of biofilm cells, bacterial swimming motility, surface hydrophobicity, auto-aggregation ability and expression of genes associated with biofilms have been evaluated to decipher possible anti-biofilm mechanisms.

## 2. Materials and Methods

### 2.1. Bacterial Strains and Culture Conditions

*V. parahaemolyticus* ATCC 17802 was purchased from the American Type Culture Collection (ATCC, Manassas, VA, USA). *V. parahaemolyticus* RIMD 2210633^Sm^ was stored in our laboratory. The other four *V. parahaemolyticus* strains (VP13, VP48, VP55, VP481) were originally isolated from marine products (shrimp and oyster) collected from a local market. All *V. parahaemolyticus* strains were cultured in Luria–Bertani (LB, Hope Bio-Technology, Qingdao, China) broth containing 3% NaCl at 37 °C with continuous 180 revolutions/min shaking. After incubation, the culture was centrifugated (5000× *g*, 5 min, 4 °C), washed with PBS and adjusted to an OD_600 nm_ of 0.5 (equivalenting to around 10^8^ CFU/mL) for later use.

### 2.2. Determination of Minimum Inhibitory Concentration (MIC)

The antimicrobial action of LEO against *V. parahaemolyticus* strains was assessed using the broth microdilution method [20]. LEO was purchased from a commercial company (Yuanye, Shanghai, China) and its main constituents include: 1.8-Cineole (30.8%), Methyl-eugenol (15.6%), a-Terpinyl acetate (14.5%), Linalool (6.1%) and Sabinene (5.3%), a-Pinene (4.9%), β-Pinene (3.2%), Terpinen-4-ol (2.8%), Eugenol (2.2%) and a-Terpineol (2.1%). One hundred microliters of LEO at concentrations ranging from 12 mg/mL to 0.0125 mg/mL were serially diluted two-fold. Additionally, 100 μL of bacterial cultures (~10^5^ CFU/mL) were added to the 96-well microtiter plate containing LEO. A microtiter spectrophotometer was used to measure the absorbance at 600 nm following a 24 h incubation period. The MIC was determined as the lowest concentration of LEO that showed no bacterial growth.

### 2.3. Determination of Subinhibitory Concentration (SIC)

To evaluate the impact of LEO on the growth of *V. parahaemolyticus*, a modified version of the previously described method was used [21]. In brief, 100 μL of *V. parahaemolyticus* ATCC 17802 suspension (~10^5^ CFU/mL) along with 100 μL of LEO solution at serial two-fold dilutions were added to a 100-well microplate to obtain final concentrations ranging from 12 mg/mL to 0.0125 mg/mL. The bacterial suspension with 3% NaCl LB was used as the control. The plate was then incubated at 37 °C for 24 h. Subsequently, bacterial growth was continuously monitored using an automatic growth curve analyzer (Bioscreen C, Oy, Finland). The SICs were defined as the concentrations of LEO that caused minimal or no inhibition to bacterial growth.

### 2.4. Biofilm Formation Assay

The crystal violet (CV) staining method was used to assess the impact of LEO on biofilm formation [22]. Overnight, bacterial suspension was adjusted to 0.5 of OD_600 nm_, with LEO at SICs, and then added to 96-well microtiter plates at 30 °C to form a biofilm. After 24 h or 48 h incubation, the media were gently removed, and wells were rinsed twice with PBS. The samples were dried for 30 min and 0.1% (*w*/*v*) CV was added into wells. Then, all samples were incubated for 20 min. The CV solution was discarded and washed. 200 μL of 33% (*v*/*v*) glacial acetic acid was added for 20 min. Finally, dissolved biofilm cells were quantified by measuring OD_630 nm_.

### 2.5. Biofilm Metabolic Activity

The metabolic activity of biofilm cells was measured using the XTT test with minor modifications [23]. The mutations formed biofilm in 96-well microtiter plates as described above. After 48 h incubation, the culture was removed and the wells were washed twice with PBS. XTT (2,3-Bis(2-methoxy-4-nitro-5-sulfophenyl)-2-Htetrazolium-5-carboxamide, Aladdin, Shanghai, China) (1 mg/mL, prepared in PBS) and menadione solution (1 mM in acetone) was prepared as the working solution. In the experiment, XTT and menadione were in a ratio of 12.5:1. Later, 200 μL PBS and 27 μL of working solution were added to the wells. The plate was placed in the dark. After 4 h, OD _490 nm_ was determined with a microplate reader.

### 2.6. Visualization of Biofilm

The biofilm was formed on a sterile microscope cover glass (φ 8 mm) in a 24-well microtiter plate. All samples were placed at 30 °C. After 48 h, the glass coverslips were washed to remove unattached cells. Field emission scanning electron microscope (FE-SEM) and fluorescence microscope assay were determined [24]. FE-SEM observation was performed to study biofilm formation as described previously. A total of 2.5% glutaraldehyde was used to fix the biofilm cells on the coverslips at 4 °C for 12 h. All samples were washed with PBS and dehydrated in a graded ethanol series. After being air dried, the samples were sputter-coated with gold under vacuum and visualized with FE-SEM (JSM-7800 F, JEOL, Japan).

Meanwhile, it was decided to test the viability of biofilm cells using SYTO9/propidium iodide (PI) staining. *V. parahaemolyticus* formed a biofilm on coverslips and was then washed. Afterward, biofilm cells were stained with SYTO 9 (5 μM) and PI (30 μM) for 20 min in the dark. At last, biofilm cells were washed with PBS and observed under a fluorescence microscope (Revole, Echo, CA, USA).

### 2.7. Swimming Motility Assay

According to Guo et al.’s protocol [25], the swimming motility of the mutations was measured. LB broth was added with 0.3% agar and 3% NaCl as the swimming semisolid medium. After being dried for 1 h, 5 μL of suspension was seeded onto the swimming semisolid agar. After 37 °C incubation, the diameters were recorded and analyzed using ImageJ software. Furthermore, the movement areas were captured with an auto-colony automatic counter (Interscience, Scan 4000, Puycapel, Cantal, France).

### 2.8. Raman Spectroscopy Analysis

The biochemical profiles of *V. parahaemolyticus* biofilms were measured using Raman spectroscopy [26]. *V. parahaemolyticus* ATCC 17802 was cultured in 24-well plates containing coverslips (ϕ 8 mm) inside at 30 °C for 48 h to form a biofilm. The Raman spectrum was recorded with a laser micro-Raman spectrometer (LabRAM HR Evolution, HORIBA Scientific, Paris, France). A diode laser at 532× nm and 100× objective was used for all Raman experiments. The Raman shift was recorded, ranging from 400 to 3200 cm^−1^. Preprocessing of Raman preliminary data was carried out using the LabSpec 6 software.

### 2.9. The Cell Surface Hydrophobicity (CSH) Assay

Bacterial adhesion to hydrocarbons (BATH) was performed using xylene [27]. Xylene is more hydrophobic than other hydrocarbons and so was selected in the experiment. Bacterial cultures were centrifuged and resuspended to maintain an OD 600 nm (A_0_) between 0.4 and 0.6. Xylene and bacterial suspension were mixed at a rate of 1:4 and vortexed for 2 min. The mixtures were left to sit for 20 min. Afterward, the aqueous phase was removed to measure the OD (Ai) of the cells remaining in the suspension. Hydrophobicity was calculated using the following formula: hydrophobicity (%) = [(A_0_ – A_i_)/A_0_] × 100.

### 2.10. Auto-Aggregation Abilities Assays

According to Ling et al., the effect of LEO on auto-aggregation was measured [27]. *V. parahaemolyticus* ATCC 17802 suspension was harvested, washed and resuspended with PBS to an OD_600 nm_ (A_0_) of approximately 0.5. Then, the suspension was transferred to tubes. After 20 h, OD_600 nm_ (A) was determined. The auto-aggregation (Aag) was calculated with the following formula: Aag% = (A_0_ – A)/A × 100%.

### 2.11. Biofilm Formation on Stainless-Steel (SS) Coupons, Glass and Food-Grade Silicone

The biofilms on SS coupons, glass and food-grade silicone were as described previously, with minor modifications [28]. Briefly, SS coupons (5 × 2 × 0.1 cm, type: 304), glass (5 × 2 × 0.1 cm) and food-grade silicone (5 × 2 × 0.1 cm) were completely submerged into 50 mL tubes. The bacterial cultures were diluted to 1:50 and then inoculated into the tubes and grown at 30 °C. Following 48 h incubation, all SS coupons, glass and food-grade silicone were removed from the bacterial culture and rinsed twice with PBS. Afterwards, each sample was transferred to a new tube containing PBS and sterile glass beads, and vortexed for 3 min. Enumerations of *V. parahaemolyticus* biofilms were determined using serial dilutions in PBS and were spread on 3% NaCl LB agar at 37 °C overnight.

### 2.12. Cytotoxicity Analysis

The murine macrophage cell line, RAW 264.7 and human colon adenocarcinoma cell line, Caco-2 were used in the study. The cytotoxicity assay of LEO was detected using the CCK8 method (Beyotime Biotechnology, Shanghai, China). RAW 264.7 and Caco-2 cells were seeded in 96-well plate including DMEM with 10% fetal bovine serum. All cells were (10^5^ cells per well) grown at 37 °C and 5% CO_2_ for 24 h. LEO at SICs were added for 12 h. The culture was eliminated. Meanwhile, 100 μL DMEM and 10 μL CKK-8 solution were added. After 1 h, OD_450 nm_ was measured.

### 2.13. Real-Time Quantitative Polymerase Chain Reaction (RT-qPCR) Assay

The effect of LEO on the transcription of *V. parahaemolyticus* biofilm genes was measured using RT-qPCR. The total RNA was extracted from log-phase *V. parahaemolyticus* suspension, with or without LEO, using a SteadyPure Universal RNA Extraction Kit (AG, Changsha, Hunan, China). The total RNA was reverse transcribed into cDNA with an Evo M-MLV RT Kit (AG, Changsha, Hunan, China). Table 1 includes a list of the primers used for RT-qPCR. RT-qPCR reactions were carried out with a SYBR Green Premix Pro Taq HS qPCR Kit (AG, Changsha, Hunan, China). The genes were determined using the cycle threshold (Ct). The comparative threshold cycle (2^−ΔΔCT^) method was used to calculate relative quantity of gene expression. The 16 S rRNA gene was served as a reference gene for data normalization.

### 2.14. Statistical Analysis

All treatments were conducted in triplicate. Data were presented as the mean ± SD. Differences were analyzed using one-way analysis of variance (ANOVA) using SPSS 19.0. Significance was indicated by * (*p* < 0.05), ** (*p* < 0.01) and *** (*p* < 0.001).

## 3. Results

### 3.1. MICs and SICs of LEO against V. parahaemolyticus

**Figure 1:**
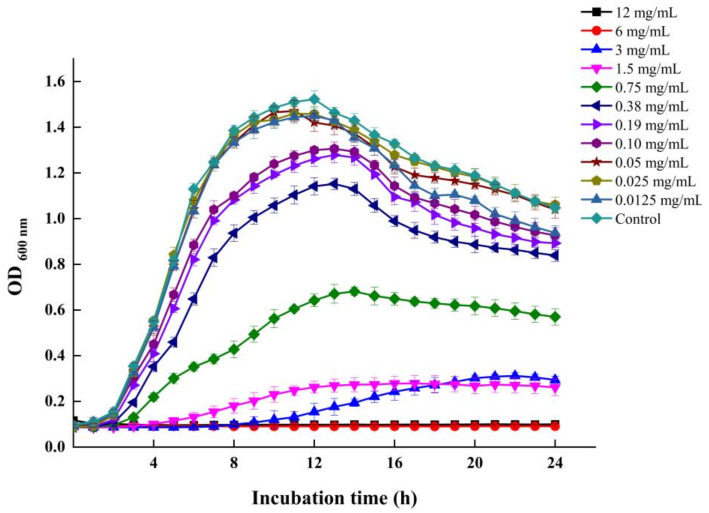
The growth curve of *V. parahaemolyticus* ATCC 17802 in the presence of various concentrations of LEO.

The MICs of LEO against the five examined *V. parahaemolyticus* strains ranged from 3 to 12 mg/mL (Table 2). The VP 13 strain showed the greatest sensitivity to LEO (MIC = 3 mg/mL). The MICs of LEO against *V. parahaemolyticus* ATCC 17802 and RIMD 2210633^Sm^ were 6 mg/mL. *V. parahaemolyticus* ATCC 17802 was selected for further experiments. Figure 1 depicts the growth curve of *V. parahaemolyticus* ATCC 17802 with various concentrations of LEO. LEO at concentrations lower than 0.05 mg/mL had minimal inhibitory or no inhibitory effect on the growth of *V. parahaemolyticus*. Therefore, 0.05, 0.025 and 0.0125 mg/mL were chosen as SICs for further investigation.

### 3.2. LEO Inhibited Biofilm Formation on Microtiter Plates

**Figure 2:**
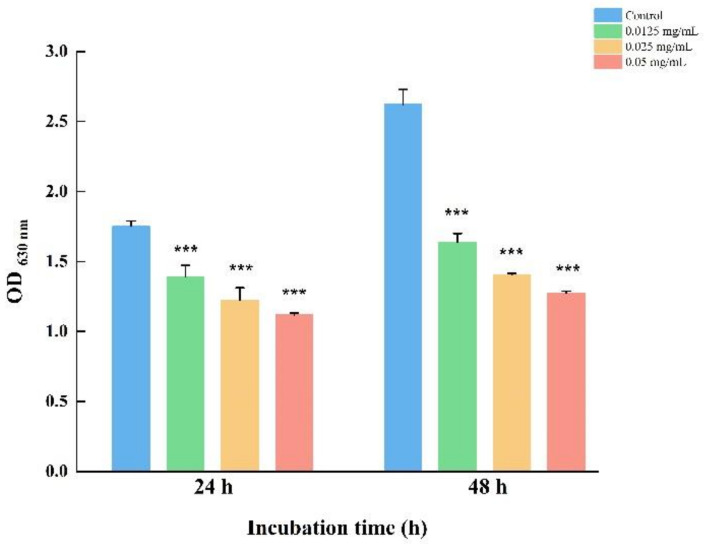
Inhibition of *V. parahaemolyticus* ATCC 17802 biofilm formation by LEO at SICs. *** *p* < 0.001 versus the control.

As shown in Figure 2, LEO at SICs demonstrated a concentration-dependent inhibitory effect on *V. parahaemolyticus* biofilms. LEO obviously reduced the biofilm by 36.05%, 30.00% and 20.39% at doses of 0.05, 0.025 and 0.0125 mg/mL. Meanwhile, the biofilm formation was inhibited by 51.40%, 46.42% and 37.52%, respectively, following incubation for 48 h (Figure 2).

### 3.3. LEO Decreased Biofilm Metabolic Activity

**Figure 3:**
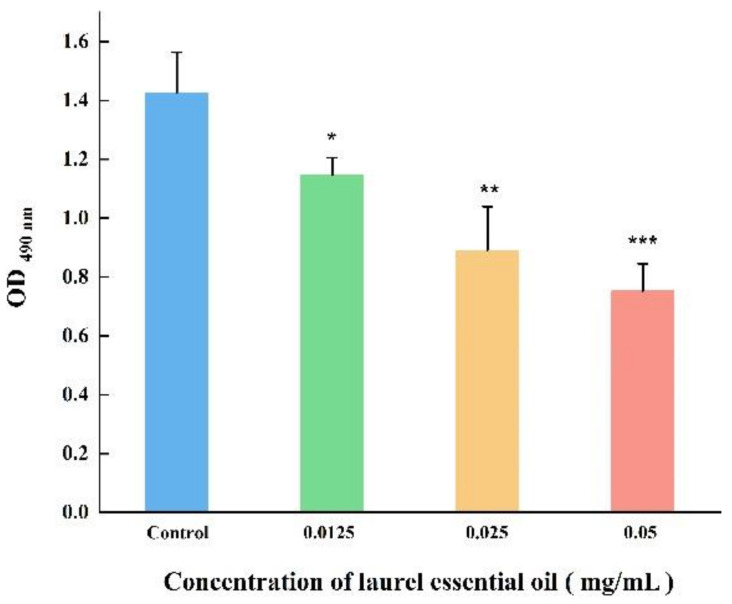
The metabolic activity of *V. parahaemolyticus* ATCC 17802 cells within biofilm. *** *p* < 0.001, ** *p* < 0.01 and * *p* < 0.05 versus the control.

In order to explore the effect of LEO on the metabolic activity of *V. parahaemolyticus* cells within biofilms, an XTT assay was performed. As shown in Figure 3, LEO at SICs obviously impaired the biofilm’s metabolic activity. At concentrations of 0.05, 0.025 and 0.0125 mg/mL, LEO reduced the metabolic activity of biofilm cells by 47.26%, 37.56% and 19.62%, respectively, compared to the control.

### 3.4. LEO Impaired V. parahaemolyticus Biofilm Structure

**Figure 4:**
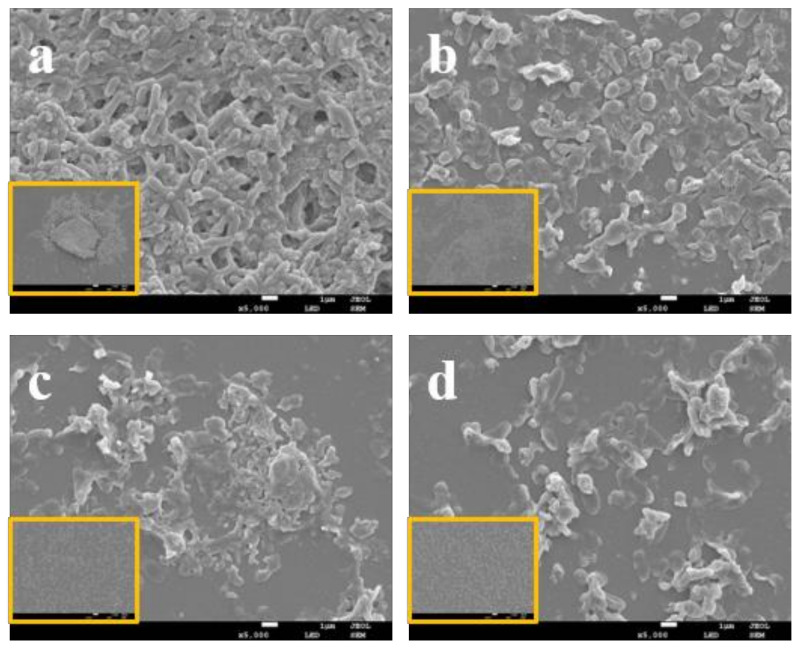
FE-SEM images of biofilm of *V. parahaemolyticus* ATCC 17802 treated with LEO at 0 (**a**), 0.0125 (**b**), 0.025 (**c**) or 0.05 mg/mL (**d**). (The large image, 5000× magnification and images in orange boxes, 500× magnification).

**Figure 5:**
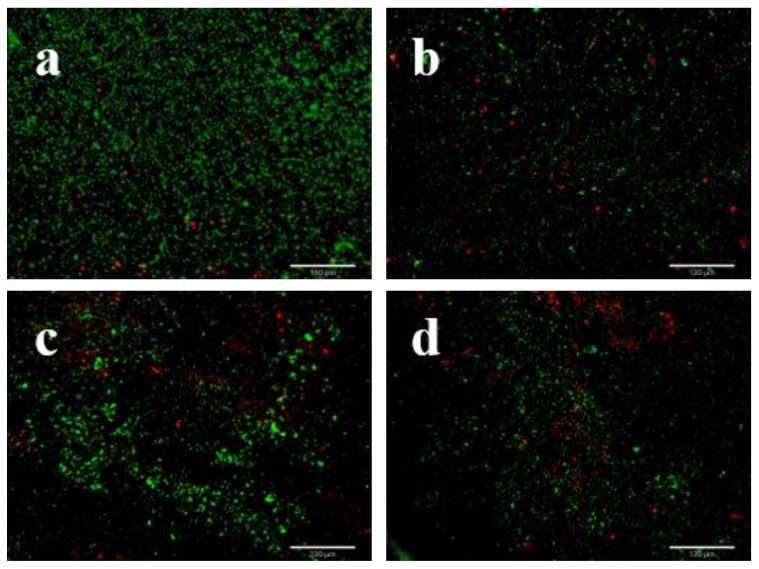
Fluorescence microscopy images of *V. parahaemolyticus* ATCC 17802 biofilm formed in the presence of LEO at concentrations of 0 (**a**), 0.0125 (**b**), 0.025 (**c**) or 0.05 mg/mL (**d**).

The effect of LEO on *V. parahaemolyticus* ATCC 17802 biofilm structure was determined by FE-SEM. Compared to the control, FE-SEM images showed that biofilm biomass at SICs of LEO significantly decreased (Figure 4). The control group formed a thick biofilm with complex self-assembled architecture with plenty of extracellular matrix (Figure 4a). In contrast, the biofilms formed under LEO treatment were less dense and showed reduced extracellular matrix (Figure 4b–d). We also applied fluorescence staining to observe viable or dead cells in the biofilms. Viable cells were stained green and dead cells red. As shown in Figure 5a, biofilm cells without LEO treatment were almost all green, which indicated that viable cells far outnumbered dead cells. Compared to the control, more red fluorescence and less green fluorescence were observed in the presence of LEO. Figure 5b–d demonstrate that the number of viable cells decreased with the increasing concentrations of LEO. Above all, FE-SEM and fluorescence microscope indicated that LEO not only interfered with the biofilm structure and biofilm biomass, but also affected cell viability.

### 3.5. LEO Inhibited Bacterial Motility

**Figure 6:**
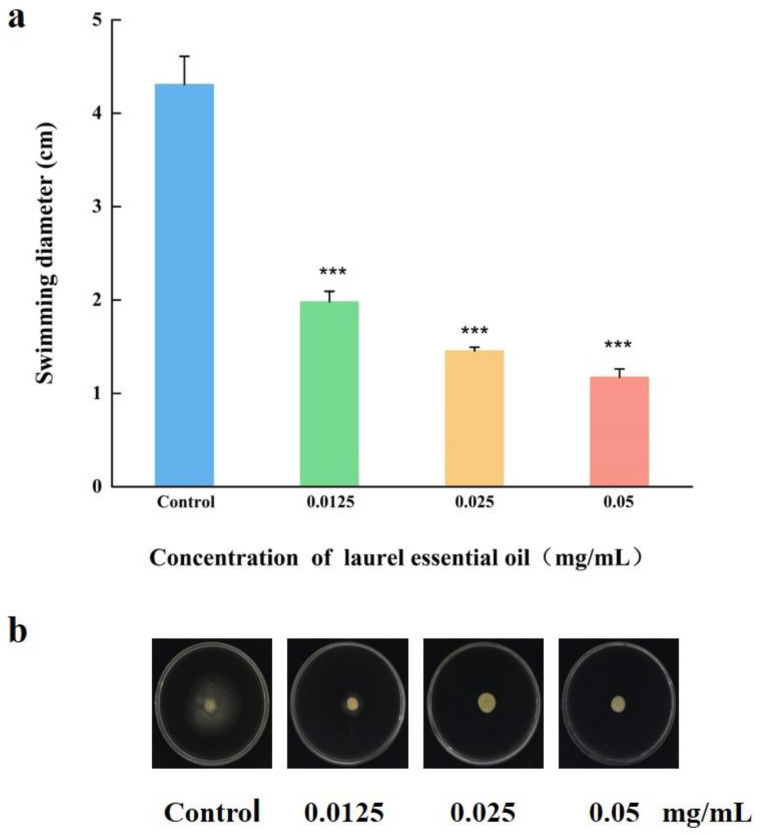
Inhibitory effects of LEO at SICs on swimming motility of *V. parahaemolyticus* ATCC 17802 (**a**). Images were captured (**b**). *** *p* < 0.001 versus the control.

The flagella-mediated motility plays a crucial role in bacterial initial attachment, which is an initial step in biofilm formation. The single polar flagellum is responsible for *V. parahaemolyticus* swimming in the liquid. In our study, the swimming motility was examined on a semisolid medium. Compared to the control, the swimming areas of *V. parahaemolyticus* treated with LEO were significantly decreased. As displayed in Figure 6, the diameter of the swimming area of untreated *V. parahaemolyticus* was 4.31 cm^−1^, whereas that of the treated group (0.05, 0.025 and 0.0125 mg/mL) was 1.17, 1.46 and 1.98 cm^−1^, respectively.

### 3.6. LEO Modified V. parahaemolyticus Biofilm Composition

**Figure 7:**
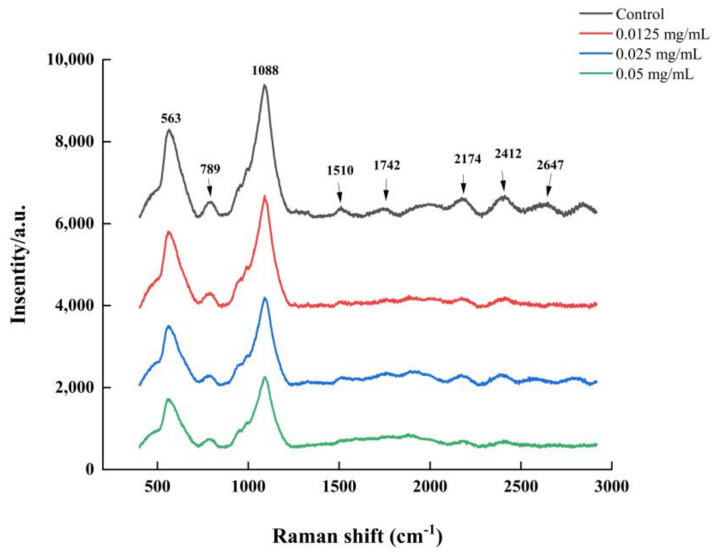
Raman spectroscopy of *V. parahaemolyticus* biofilm formed under LEO treatment.

The Raman spectroscopy was carried out for biofilms formed on coverslips. Raman spectral features of *V. parahaemolyticus* biofilms were presented in Figure 7. According to previous studies, the dominant peak assignments of the bands were summarized. The major peaks were at 563, 789, 1088, 2174 and 2412 cm^−1^. Several other minor peaks were found at 1510, 1752 and 2647 cm^−1^. Furthermore, typical vibrational Raman bands of carbohydrates, proteins, carbohydrates and nucleic acid could be observed in the Raman spectra of *V. parahaemolyticus* biofilms. The distinct Raman bands were considered as carbohydrates: 563 cm^−1^ was attributed to C-O-C glycosidic ring deformation vibration, and 789 cm^−1^ was assigned to the O-P-O stretch of DNA [29]. The Raman bands at 1088 cm^−1^ and 1742 cm^−1^ belonged to carbohydrates and lipids, respectively [30]. The peak at 1510 cm^−1^ was assigned to C=C stretching of carotenoids [31]. The Raman bands at 2102–2422 cm^−1^ were due to a mix of C=C or C=O bond vibrations in fatty acids, DNA strand bonds and amide bonds in proteins [32]. Compared to the control, a *V. parahaemolyticus* biofilm treated with LEO presented decreased Raman peak intensities. In particular, Raman peaks at 563 and 1088 cm^−1^ corresponding to carbohydrates showed the greatest reduction. The peak intensities of 2174 and 2412 cm^−1^ also exhibited a decreasing trend. The Raman peaks at 1510, 1742 and 2647 cm^−1^ disappeared. In addition, the peak intensity at 789 cm^−1^ remained unchanged.

### 3.7. LEO Decreased CSH and Auto-Aggregation Abilities of V. parahaemolyticus

**Figure 8:**
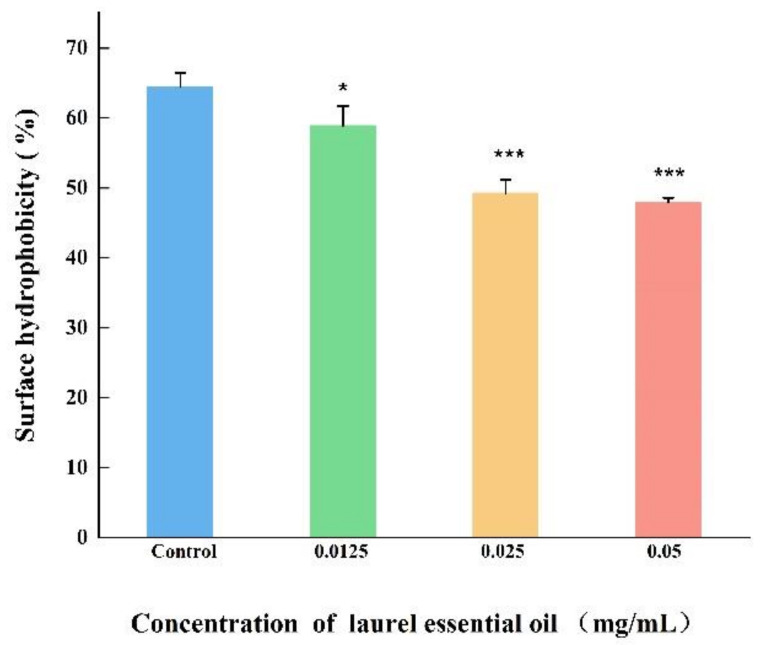
The effect of LEO on surface hydrophobicity of *V. parahaemolyticus* ATCC 17802. *** *p* < 0.001 and * *p* < 0.05 versus the control.

**Figure 9:**
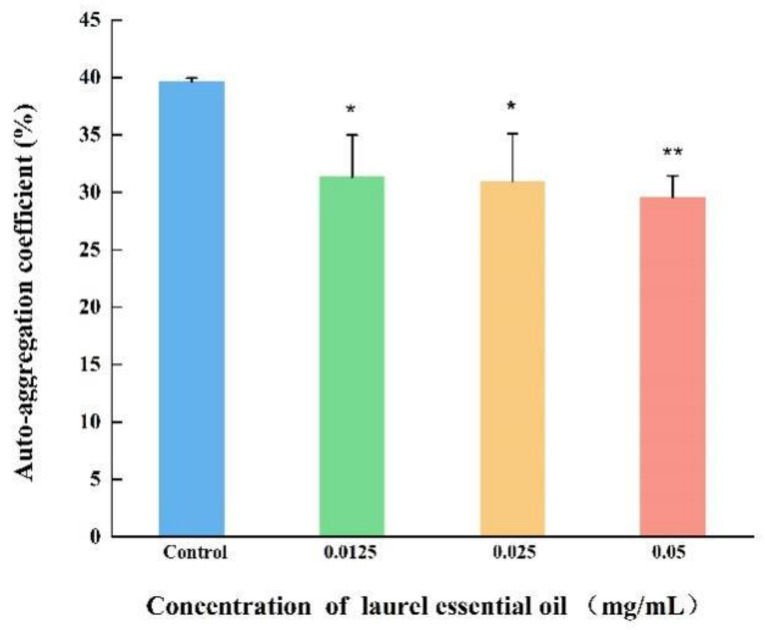
The effect of LEO on auto-aggregation coefficient of *V. parahaemolyticus* ATCC 17802. ** *p* < 0.01 and * *p* < 0.05 versus the control.

CSH and aggregation abilities are crucial factors for *V. parahaemolyticus* to adhere to various surfaces. As displayed in Figure 8, *V. parahaemolyticus* untreated with LEO exhibited high CSH. CSH of *V. parahaemolyticus*, treatment with LEO (0.05, 0.025 and 0.0125 mg/mL) were reduced by 16.63%, 15.26% and 5.59%, respectively, compared to the control. Similarly, a significant inhibition in aggregation ability was also observed after LEO treatment (Figure 9). Compared to the control, auto-aggregation of *V. parahaemolyticus* treated with LEO at concentrations of 0.05, 0.025 and 0.0125 mg/mL was inhibited by 10.07%, 8.66% and 8.29%, respectively.

### 3.8. LEO Reduced Biofilm Cells on Food Contact Surfaces

**Figure 10:**
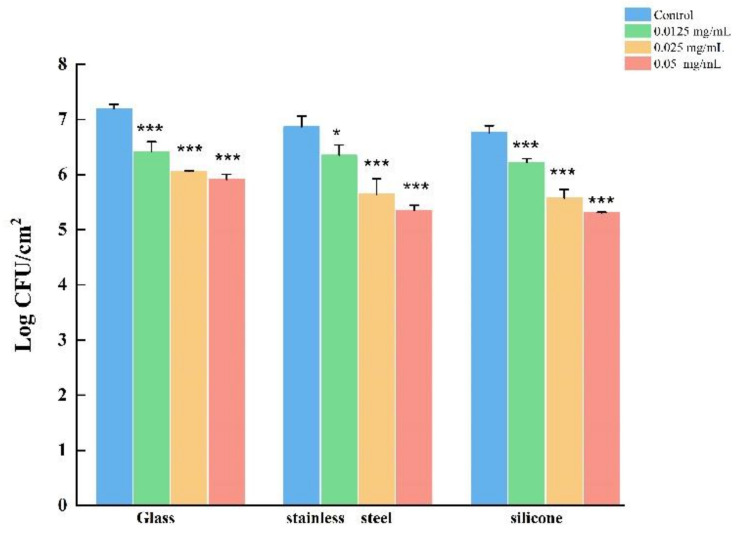
The effects of LEO on the adhesion of *V. parahaemolyticus* ATCC 17802 to different food-contact surfaces. *** *p* < 0.01 and * *p* < 0.05 versus the control.

The effect of LEO on *V. parahaemolyticus* biofilm cells on food-contact surfaces (SS, silicone and glass) was shown in Figure 10. The initial biofilm cell count on the glass surface was 7.19 log CFU/cm^2^, and was 6.87 and 6.76 log CFU/cm^2^ on the SS and silicone surfaces, respectively. Compared to the control, LEO treatment (0.05, 0.025 and 0.0125 mg/mL) reduced the cell numbers on glass by 17.92%, 15.97% and 10.83%, respectively. The *V. parahaemolyticus* cell count on the SS surface was reduced by 22.13%, 17.90% and 7.60%, respectively. Similarly, Figure 10 reveals that *V. parahaemolyticus* count in the biofilm cells of the silicone surface was reduced by 21.45%, 17.60% and 8.14%, respectively, with LEO treatment (0.05, 0.025 and 0.0125 mg/mL).

### 3.9. LEO Was Non-Cytotoxic to Cells

**Figure 11:**
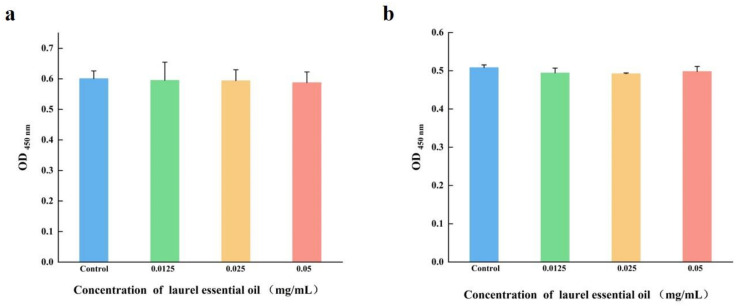
The effect of LEO on Caco-2 (**a**) and RAW 264.7 (**b**) cell cytotoxicity.

The effect of LEO on cell cytotoxicity is shown in Figure 11. After LEO treatment, the Caco-2 cell activity was 99.10%, 98.88% and 97.78% in the presence of LEO (0.05, 0.025 and 0.0125 mg/mL), compared to the control (Figure 11a). Similarly, Figure 11b illustrates that the RAW 264.7 cell activity was 97.25%, 96.87% and 97.99% with LEO treatment (0.05, 0.025 and 0.0125 mg/mL). Above all, LEO at SICs was non-cytotoxic to RAW 264.7 and Caco-2 cells. These results might provide theoretical support that LEO at SICs are safe in food and food industries.

### 3.10. LEO Down-Regulated Expression of Genes Related to Biofilm Formation

**Figure 12:**
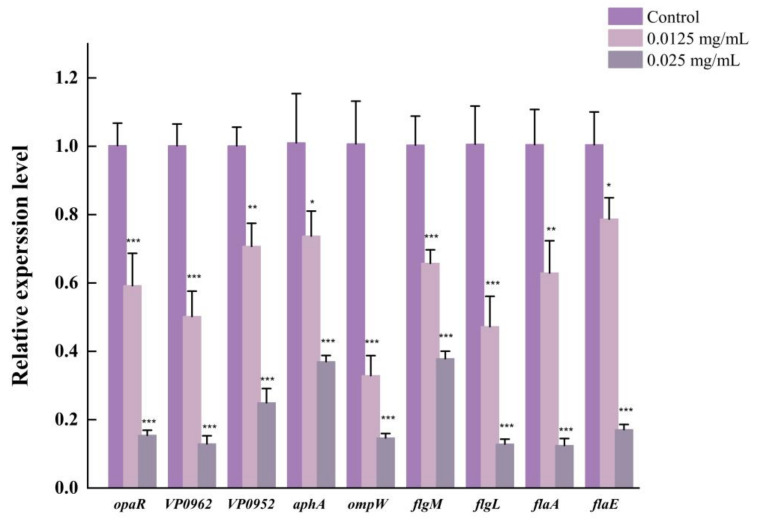
The effect of LEO on transcript level of genes related to biofilm formation of *V. parahaemolyticus* ATCC17802. *** *p* < 0.001, ** *p* < 0.01 and * *p* < 0.05 versus the control.

The impact of LEO on the expression of genes involved in biofilm formation was investigated using RT-qPCR. Out of the nine genes analyzed, structural genes of *V. parahaemolyticus* flagellum (*flgM*, *flgL*, *flaE* and *flaA*), quorum sensing production regulators (*aphA* and *opaR*) and other biofilm-formation-related genes (*ompW, VP0952* and *VP0962*) were significantly downregulated after LEO treatment (Figure 12). As displayed in Figure 12, LEO at 0.025 mg/mL downregulated the transcription of *aphA* and *opaR* genes by 2.73-fold and 6.67-fold. LEO treatment decreased the expressions of *flgM*, *flgL*, *flaE* and *flaA* genes by 2.63-fold, 7.69-fold, 5.88-fold and 7.69-fold, respectively. The transcription levels of *ompW*, *VP0952* and *VP0962* were repressed by 6.67-fold, 4-fold and 7.69-fold, respectively.

## 4. Discussion

It is reported that *V. parahaemolyticus* is one of the primary causes of foodborne infections in many countries, especially in coastal areas [33]. *V. parahaemolyticus* can colonize biological or non-biological surfaces to form biofilms. Biofilms can secrete extracellular polymeric substances (EPS), which increase the difficulty of disinfection and eradication [34]. In addition, biofilm cells show increased resistance to antibiotics, thereby rendering some antibiotics ineffective against biofilm-caused infections. Hence, exploring natural and safe strategies to create a cost-effective and safe substance to resist biofilms is imperative for the seafood industry. Previous study has proved that EOs are suitable alternatives for antibiotics [35]. Therefore, LEO was chosen in this study. LEO is used as a flavoring in food and is also used in the pharmaceutical industry because of its anti-inflammatory and antioxidant properties [18]. Moreover, LEO has been investigated for its ability to interrupt bacterial and fungal growth. As previously described, LEO has antimicrobial activity against *Yersinia enterocolitica* and *Escherichia coli*. It also has antifungal activity towards Penicillium digitatum, Monilinia laxa and Botrytis cinerea [36]. Nevertheless, the efficacy of LEO against *V. parahaemolyticus* biofilms has not been studied before.

Our study suggests that LEO at SICs concentrations could effectively control *V. parahaemolyticus* biofilm formation. We used SICs to test the anti-biofilm potential of LEO to avoid selective pressure on growth, which may lead to the development of antibiotic resistance [37]. Our results illustrate that LEO at SICs obviously inhibits biofilm biomass and metabolic activity. Similarly, Guo et al. (2019) indicated that essential oil from Citrus Changshan-huyou decreased the biomass and metabolic activity of *Listeria monocytogenes* biofilms, thereby destroying preformed biofilms [38]. The process of biofilm formation is dynamic and begins with free-floating microorganisms attaching to surfaces, thereby forming the characteristic biofilm structure [39]. FE-SEM images confirm that biofilm structure is loose and discrete after LEO treatment. The results demonstrate that LEO at SICs retarded bacterial cells’ attachment, which may lead to significantly reduced biofilm formation. Our findings are consistent with previous studies, in that some anti-biofilm agents could disrupt biofilm architecture and loosen attached microcolonies [40,41].

Bacteria could attach to various biotic and abiotic surfaces partly owing to flagellar-mediated motility, which successfully facilitates the initial adhesion and positively modulates biofilm formation. In this study, LEO at SICs significantly repressed swimming motility and further downregulated the expression of genes related to motility, including *flgM*, *flgL*, *flaA* and *flaE*. Cao et al. reported that citral inhibited the swimming ability of *V. parahaemolyticus* and also downregulated the transcriptions of genes related to motility [42]. In addition, thymoquinone effectively inhibited biofilm formation by reducing the motility and downregulating the transcriptions of *flgM*, *flgL* and *flaA* in *V. parahaemolyticus* [25]. EPS can hold the bacterial cells together and make the biofilm firmly attach to surfaces, which is important for biofilm maturation [43]. EPS are mainly composed of polysaccharides, lipids, nucleic acids and proteins. Among them, polysaccharides are the most abundant component [24]. We used laser micro-Raman spectrometer to examine biochemical compositions in *V. parahaemolyticus* biofilms. Our study indicated that two maximum absorption peaks were at 563 and 1088 cm^−1^, which were assigned as polysaccharides, which confirmed that exopolysaccharides accounted for the majority of biofilm components. Moreover, LEO treatment led to a decreased amount of polysaccharides and other biochemical compositions (lipids, proteins and nucleic acids), which could be assessed using the lower height of corresponding peaks.

CSH is an important factor in the cell attachment stage, aggregation and colony assembly, thereby affecting biofilm formation. Furthermore, aggregation ability plays an essential role that interferes with the adhesion of pathogenic bacteria to various surfaces through competitive exclusion, leading to biofilm development and stability of the bacterial biofilm colonies [44]. Ling et al. have described a good correlation between biofilms, CSH and auto-aggregation in *Cronobacter sakazakii* [27]. In their study, the *grxB* deletion mutant significantly inhibited biofilm formation by decreasing CSH and auto-aggregation. A significant positive correlation was found between auto-aggregation and biofilm formation in Myroides odoratus [45]. These findings suggest that that LEO reduced biofilm formation in *V. parahaemolyticus* partly due to reduced CSH and auto-aggregation.

## 5. Conclusions

In summary, LEO at SICs decreased biofilm biomass, disrupted biofilm architecture, reduced metabolic activity and changed biochemical composition of biofilms of *V. parahaemolyticus*. The reduced biofilms caused by LEO was associated with decreased bacterial swimming motility, CSH, auto-aggregation and decreased expression of biofilm-related genes of *V. parahaemolyticus*. These findings suggest that LEO could be potentially developed as an alternative strategy to control *V. parahaemolyticus* biofilms in food industries.

## Figures and Tables

**Table 1:** Primers used in RT-qPCR.

Target Gene	Sequence of Primers (5′–3′)
16S rRNA	F: GCCTTCGGGAACTCTGAGACAG
	R: GCTCGTTGCGGGACTTAACCCAA
*opaR*	F: AGGGCATCGTTACCCAATC
	R: TAAGTCAACATAGTCCGCATC
*VP0962*	F: GACCAAGACCCAGTGAGA
	R: GGTAAAGCCAGCAAAGTT
*VP0952*	F: TATGATGGTGTTTGGTGC
	R: TGTTTTTCTGAGCGTTTC
*aphA*	F: ACACCCAACCGTTCGTGATG
	R: GTTGAAGGCGTTGCGTAGTAAG
*ompW*	F: TCGTGTCACCAAGTGTTTTCG
	R: CGTGGCTGAATGGTGTTGC
*flgM*	F: AGAAATGAAATCGCTACCAGT
	R: GGAATTGCATAATTGCCTTA
*flgL*	F: CGTCAGCGTCCACCACTT
	R: GCGGCTCTGACTTACTGCTA
*flaA*	F: CGGACTAAACCGTATCGCTGAAA
	R: GGCTGCCCATAGAAAGCATTACA
*flaE*	F: ACAGTGCGGATAGCCAGTA
	R: CTTTGAGTAGCGTCTCGTTT

**Table 2:** MICs of LEO for *V. parahaemolyticus* strains.

Strain	Origin	MICs(mg/mL)
ATCC 17802	Shirasu food poisoning	6
RIMD 2210633^Sm^	Pandemic strain	6
VP 13	Seafood	3
VP 48	Seafood	12
VP 55	Seafood	6
VP 481	Seafood	12

## Data Availability

Data are contained within the article.
